# Physicochemical Properties of Cold‐Press Sesame (*Sesamum indicum* L.) Seed Oils and Their Defatted Residues

**DOI:** 10.1002/fsn3.71168

**Published:** 2026-01-02

**Authors:** Sazna Fariz, Fahmidha Halaldeen, Terrance Madhujith, Nazrim Marikkar, Muneeb M. Musthafa, Mohammed Arshad, Abdul Aziz Al Kheraif

**Affiliations:** ^1^ National Institute of Fundamental Studies Kandy Sri Lanka; ^2^ Postgraduate Institute of Agriculture University of Peradeniya Peradeniya Sri Lanka; ^3^ Department of Biosystem Technology South Eastern University of Sri Lanka, University Park Oluvil Sri Lanka; ^4^ Dental Biomaterials Research Chair, Dental Health Department, College of Applied Medical Sciences King Saud University Riyadh Saudi Arabia

**Keywords:** ANKSE3, defatted residue, DSC, proximate composition, sesame oil, UMA

## Abstract

This study aimed to examine the physicochemical properties, fatty acid composition, differential scanning calorimetric (DSC) profile, and Fourier transform infrared (FTIR) spectral properties of sesame oils extracted from ANKSE3 and UMA cultivars of Sri Lanka. Cold press extraction of the raw seed yielded good quality oil and edible grade defatted residues. Proximate compositional analysis showed that fat was the main constituent of the raw seeds regardless of the cultivar, while protein was the predominant constituent of the defatted residues. The color intensity of the oil of the UMA cultivar was stronger than that of ANKSE3. High iodine values and lower saponification values were observed in the oils of both cultivars. Both oils contained approximately 85% unsaturated fatty acids (USFAs), and 15% saturated fatty acids (SFAs), with oleic and linoleic acids being the most abundant. The existence of triacylglycerols (TAGs) of varying melting temperatures was clearly exhibited by distinct exothermic and endothermic peaks of the DSC curves. The occurrence of organic functional groups like alkanes, alkenes, fatty esters, etc. was clearly demonstrated by FTIR spectra of sesame oils. The findings highlighted the superiority of sesame oils of ANKSE3 and UMA as nutritious oils due to their high content of USFAs, and defatted residues of sesame as a good source of alternative vegetable protein for value‐added product formulations.

## Graphical abstract

The findings highlighted the superiority of sesame as a nutritious oil due to its high content of USFAs, and defatted residues of sesame as a good source of alternative vegetable protein for value added product formulations.

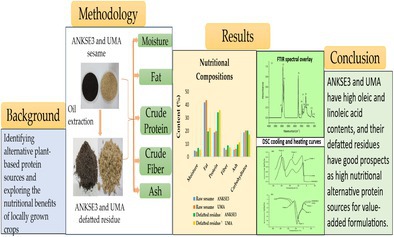

## 1 Introduction

Oil is one of the major concentrated energy sources essential in the human diet. Studies on exploring newer and underutilized plants for oil production have gained increasing interest due to the rising demand for vegetable oils (Raihana et al. 2015). Assessment of both quality and quantity of various fruit seeds has increasingly become a focal point of research in the oilseed industry. The types of seed, their size, and shape are some of the factors that affect oil quantity, while their quality characteristics differ mainly based on the method of processing, packaging, fatty acid and triacylglycerol compositions (Ndangui et al. 2010). Apart from their nutritional value in the human diet, oils are also useful as ingredients in novel cosmetic formulations to improve texture, flavor, and other sensorial properties.

Among oils and fats, there are different sub‐classes, namely vegetable oils, animal depot fats, ruminant milk fats, and marine oils. They differ from one another based on compositions, physico‐chemical characteristics, thermal profile etc. They are further divided into many sub‐groups based on fatty acid and triacylglycerol compositions. For instance, lauric acid predominant oils (coconut and palm kernel oils) are referred to as lauric oils, palmitic predominant oils (palm oil and its fractions) are referred to as palmitic oils, oleic acid predominant oils (olive and canola oils) are referred to as oleic oils and so on. There has been a rising interest in seeds with nutritionally beneficial and healthier polyunsaturated fatty acids (Wijesekera et al. 2024), as well as the utilization of their byproducts with a focus on upcycling and waste management (Halaldeen et al. 2024). Owing to their nutritional value, seeds like guava, pumpkin, okra, and grape are often considered superfoods or functional foods.

Belonging to the family of Pedaliaceae, sesame seed ( *Sesamum indicum* L.) also shares certain common characteristics of some of the fruit seeds. Sesame is generally considered an oil‐rich seed, carrying nearly 40%–60% of oil. The highest sesame producers in the global market are Sudan, India, China, Myanmar, and Tanzania (Wei et al. 2022). According to some previous studies, they may contain approximately 50% fat, around 25% protein and be rich in minerals such as Fe and Ca (Rout et al. 2018). However, the chemical composition of sesame seeds might be influenced by factors such as variety, origin, color, and size of the seed. Sesame oil is an unsaturated edible oil as it contains high quantities of essential fatty acids. The main fatty acid is the monounsaturated fatty acid (MUFA) like oleic acid (35%–54%), polyunsaturated fatty acid (PUFA) like linoleic acid (39%–59%) (Hall 2003).

*S. indicum* , was introduced into arid and semi‐arid regions of Sri Lanka sometime back and is presently under cultivation on a small scale. Malee, MI, UMA, and ANKSE3 are some of the varieties developed by the Sri Lanka Department of Agriculture (Dissanayake and Jayathilaka 2021). Sri Lanka mainly cultivates sesame to obtain seeds to be used as food ingredients, but a small portion is committed to extracting edible oil. Some studies on sesame cultivation, agronomic practices, genetic diversity and plant breeding have been performed in Sri Lanka since 1990 (Dissanayake et al. 2017, 2019; Perera and Pushpakumara 2014). Rajkumar and Selvakulasingam (2019) investigated the nutritional composition of sesame seeds, Bandara et al. (2014) studied the physiochemical properties of sesame and Bopitiya and Madhujith (2013) explored the antioxidant capacity and total phenolic content of sesame. Nonetheless, several important aspects, including inter‐varietal differences of the oils from locally grown sesame cultivars with regard to their physiochemical properties, DSC thermal profiles and FTIR spectral characteristics have been overlooked. In this study, two cultivars namely ANKSE3 and UMA were selected for investigation in terms of physicochemical characteristics, fatty acid profiling, DSC thermal behavior, FTIR spectral characteristics of oils and to evaluate the proximate compositions of their defatted residues. The fact of the matter is that these two sesame types are the most widely cultivated local cultivars in Sri Lanka and their popularity among farmers is largely due to high yield and broad adaptability (Dissanayake et al. 2017, 2019). Detailed information on the nutritional composition of these two seed types, oil quality and physicochemical characteristics of the oils would be greatly beneficial for future development.

## 2 Materials and Methods

### 2.1 Sampling

#### 2.1.1 Sample Collection

Sesame seeds ( *S. indicum* L.) of two locally available cultivars, namely UMA and ANKSE3, were collected from the Seed and Planting Material Development Center (SPMDC), Bata‐atha (6.1099° N, 80.9014° E), Hambantota, Sri Lanka, between February and March 2024. The plants were cross‐checked by a Senior Taxonomist, Dr. D.S.A. Wijesundera, from the Royal Botanical Garden of Sri Lanka and voucher specimens of the species were deposited in Pelwehera farm, Dambulla, Sri Lanka.

#### 2.1.2 Sample Pre‐Treatment

Initially, pure seeds of *S. indicum* L. of the two cultivars were manually cleaned and dried in an oven (Biobase, model—BOV‐V230F, China) maintained at 55°C for 2 h. After drying, samples were kept under refrigerated conditions until further experimental work.

#### 2.1.3 Micro‐Expeller Oil Extraction

Sesame seeds were cold pressed using a micro‐oil expeller (Komet DD85 machine, Germany) to extract oil. After oil extraction, the crude oil from each cultivar was purified through gravitational filtration and defatted residues were collected separately and kept under refrigerated conditions until further analysis.

### 2.2 Physicochemical Characteristics of the Oil

Color: A Lovibond Tintometer (PFX‐I UK) was used to measure the oil's color and expressed in terms of red (R) and yellow (Y) units (Y + 5R), by following AOCS Method Cc 13b‐45 (AOCS 2017).

Iodine value (IV): IV of the oils was determined by following AOCS Method Cd 1d‐92(AOCS 2022). Initially, 10.0 mL of chloroform was added to an iodine flask containing 0.5 g of the oil sample. Subsequently, 25.0 mL of iodine solution was introduced, and the flask was kept in the dark for 1 h. After the incubation period, 10.0 mL of 10% potassium iodide solution was added, and the mixture was shaken thoroughly. Next, 75.0 mL of distilled water was added to the flask. Then, the mixture was titrated with 0.1 N sodium thiosulphate until it became nearly colorless. After adding a 1% starch solution, the titration was continued until the blue color disappeared.

$$\text{Iodine value}=\left(B-S\right)\times N\times 100/\text{Weight of oil sample}$$

where *B*—volume of Na_2_S_2_O_3_ used for the blank, *S*—volume of Na_2_S_2_O_3_ used for the oil sample, *N*—normality of Na_2_S_2_O_3_ Solution.

Saponification value (SV): SV of the oils was measured according to AOCS method Cd 3‐25. Initially, 5 g of oil sample was dissolved in 50.0 mL of 4% ethanolic KOH in a round bottom flask. The flask was then refluxed for next 30 min to ensure completion of saponification of the sample. After cooling, a few drops of phenolphthalein were added to the mixture. 0.5 N HCl was used to titrate the resultant solution until the pink color was completely disappeared.

$$\text{Saponification value}=\left(V_{B}-V_{S}\right)\times 28.05/\text{Weight of oil sample}$$

where *V*_B_—volume required for the blank; *V*_S_—volume required for the oil sample.

### 2.3 Fatty Acid Analysis

Fatty acid (FA) determination was performed following the procedure outlined by Wijesekera et al. (2024), with minor adjustments. An oil sample (0.4 g) was placed into screw‐capped glass tubes. Into this, a 4.0 mL portion of methanol, along with 0.1 mL of methanolic KOH was added. The mixture was then heated in a water bath at 60°C for 10 min and subsequently left to cool. After cooling, a 2.0 mL portion of hexane and a 4.0 mL portion of distilled water were added to the mixture, and the contents were vortexed at 2500 rpm for 10 min. Once the layers were separated, the upper layer was collected and injected into a gas chromatograph (Agilent 7890B, China) equipped with a flame ionization detector (FID). The analysis was performed using a polar capillary column (100 m × 250 μm × 0.2 μm, CP‐Sil 88 for FAME) with a column pressure of 39.512 psi. The oven temperature was set as follows: an initial temperature of 100°C for 5 min, then raised from 100°C to 180°C at a rate of 8°C per minute, followed by an increase from 180°C to 230°C at a rate of 1°C/min and finally held at 230°C for 15 min. The temperatures of the injector and detector were set to 260°C. Nitrogen was used as the carrier gas at a flow rate of 1.2863 mL/min, and the injector was operated with a split ratio of 50:1. FAs were recognized by comparing their retention times with those of standard fatty acid methyl esters, and their percentages were calculated based on their peak areas relative to the total peak areas of all detected FAs.

### 2.4 Thermal Analysis of Oil by DSC

Differential scanning calorimetric (DSC) analysis was performed using the method described by Halaldeen et al. (2024) with some adjustments. Using an aluminum T‐zero pan with a T‐zero hermetic lid, the Q200 differential scanning calorimeter (TA Instruments, USA) was employed for the analysis. At a flow rate of 50.00 mL/min, 99.9% pure nitrogen gas was utilized as the purge gas. A standard DSC aluminum pan was filled with 10–12 mg of the sample (in liquid form) and hermetically sealed. An empty hermetically sealed aluminum pan was employed as a reference. The thermal analysis was conducted using the following temperature program: an isotherm at −80°C for 1 min, followed by heating at 5°C/min to 20°C, an isotherm at 20°C for 1 min, and then cooling at 5°C/min back to −80°C.

### 2.5 FTIR Measurements

FTIR measurements of the oil samples were performed using the procedure defined by Gunarathne et al. (2022) with a few amendments. In each measurement, a 100 mg portion of KBr (FT‐IR grade, 99% trace metals basis, Sigma Aldrich) was combined with approximately 1.0 mg of oil to create a pellet. The mid‐infrared spectra, ranging from 4000–500 cm^−1^, were recorded using a FTIR Nicolet iS50 spectrometer (Thermo Nicolet, Madison, WI) equipped with a deuterated triglycine sulfate (DTGS) detector and KBr beam splitter. The spectra were obtained by co‐adding 64 scans with a resolution of 8 cm^−1^. Each spectrum was compared to a background spectrum of pure KBr, and absorbance values were recorded in four duplicates.

### 2.6 Proximate Analysis

The proximate compositional analysis of sesame seeds and defatted residues from both cultivars was conducted to evaluate moisture, total ash, crude fat, crude fiber and crude protein contents, following the procedures outlined in the AOAC International (2019) manual. Moisture content was measured using an oven (Biobase, model—BOV‐V230F, China) by drying samples at 105°C for 3 h until constant weight was achieved (AOAC Official Method 934.06). Crude fat content was determined through Soxhlet extraction with hexane (40°C–60°C) as the solvent (AOAC Official Method 948.22). The ash content was measured using the dry ashing method (AOAC Official Method 942.05), while crude protein was assessed using the micro Kjeldahl method (AOAC Official Method 970.02). The crude fiber content was analyzed by sequential digestion with sulfuric acid and sodium hydroxide solution (AOAC Official Method 962.09). The total carbohydrate content was calculated with the formula: Total carbohydrate content (%) = 100 − (Moisture % + ash % + protein % + fat % + crude fiber %).

### 2.7 Spectral Analysis

Spectral pre‐processing and qualitative analysis were carried out using the manufacturer's software (OMNIC, version 7.0 Thermo Nicolet). The raw spectra of the oils from ANKSE3 and UMA cultivars were baseline corrected and subjected to a scale normalization procedure.

### 2.8 Statistical Analysis

In this study, all chemical measurements were recorded in triplicate (*n* = 3) and data were presented as mean ± standard deviation (SD). Statistical analysis was carried out using one‐way analysis of variance (ANOVA) followed by Tukey's test, employing the Minitab 17 software package.

## 3 Results

### 3.1 Physicochemical Characteristics of *S. indicum* Oil

Comparison of color, IV, and SV of the oils obtained from both UMA and ANKSE3 cultivars is presented in Table 1. The color values of ANKSE3 and UMA cultivars were 7.45 ± 0.64 and 13.5 ± 0.14, respectively. The color values of the two oils were remarkably (*p* < 0.05) different; the UMA cultivar showed a value higher than the ANKSE3 cultivar. The results were comparable to the visual appearance of the oils, where the color of the UMA cultivar was more intense than that of the ANKSE3 cultivar. According to Table 1, the IV for ANKSE3 and UMA sesame cultivars was 105.3 ± 0 g/100 g and 116.03 ± 1.79 g/100 g, respectively. On the basis of data analysis, a remarkable difference (*p* < 0.05) was seen between the two cultivars. Overall, the ANKSE3 cultivar had a higher degree of unsaturation when compared to the UMA cultivar. According to Table 1, SV of the ANKSE3 cultivar was 186.81 ± 0.85 (mg of KOH g^−1^) while that of the UMA cultivar was 187.94 ± 1.45 (mg of KOH g^−1^). Nonetheless, no remarkable (*p* > 0.05) difference was noticed between them.

**TABLE 1:** Color, iodine value, and saponification number of oil from ANKSE3 and UMA cultivars.

Variety	Iodine value (g I_2_/100g)	Saponification number (mg KOH/g)	Color Y + 5R
ANKSE3 cultivar	105.30^a^ ± 0.00	186.81^a^ ± 0.85	7.45^a^ ± 0.64
UMA cultivar	116.03^b^ ± 1.79	187.94^a^ ± 1.45	13.50^b^ ± 0.14

*Note:* Each value in the table represents the mean of three replicates ± standard deviation. The means within each column bearing different superscripts are significantly (*p* < 0.05) different.

Abbreviations: IV, iodine value; SV, saponification value.

### 3.2 FA Profile of *S. indicum* Oil

**FIGURE 1:**
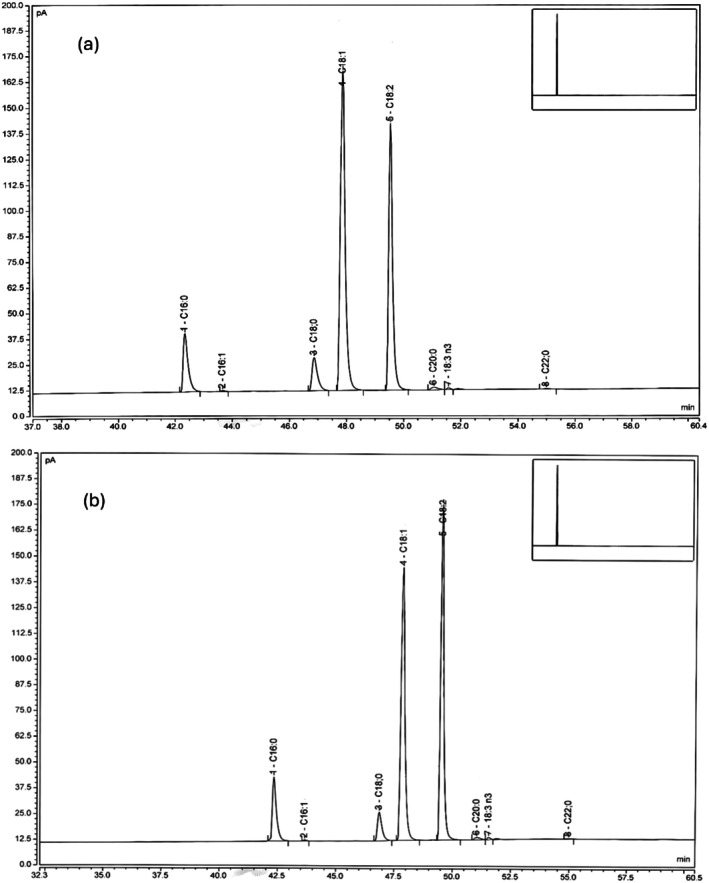
Fatty acid chromatograms of oils from ANKSE3 (a) and UMA (b) cultivars.

Figure 1a,b display the GLC chromatograms of fatty acid methyl esters of oils from ANKSE3 and UMA cultivars. The quantitative distribution of individual FAs of sesame oils of the two cultivars is compared as shown in Table 2. The oils extracted from the two cultivars were found to contain predominantly USFAs, with total contents of 84.52% and 84.66%, respectively. Oleic acid was the most dominant unsaturated FA in both cultivars; ANKSE3 contained a significantly (*p* < 0.05) higher proportion of oleic acid (50.07%) when compared to UMA (42.31%). In both of these cultivars, the second most predominant FA was linoleic acid. The proportion of linoleic acid in the UMA cultivar (42.04%) was comparatively higher (*p* < 0.05) than that of the ANKSE3 cultivar (34.17%). Other unsaturated FAs such as linolenic and palmitoleic acids were found to exist in minute quantities in both cultivars; linolenic acid contents of ANKSE3 and UMA cultivars were 0.18% ± 0.03% and 0.22% ± 0.03%, respectively, with a significant (*p* < 0.05) difference between them. The proportions of palmitoleic acid in oils of ANKSE3 and UMA were 0.10% ± 0.0% and 0.09% ± 0.0%, respectively, with no remarkable (*p* > 0.05) difference between them. Palmitic, stearic, arachidic, and behenic acids were the saturated acids in the two cultivars; the total saturated FA contents of ANKSE3 and UMA were 15.41% and 15.36%, respectively. Being the most abundant saturated FA, palmitic acid was present at 8.93% ± 0.40% in the ANKSE3 cultivar while at 9.85% ± 0.38% in the UMA cultivar. Likewise, the proportions of stearic acid in both ANKSE3 and UMA cultivars were 5.91% ± 0.04% and 4.95% ± 0.00%, respectively. Nonetheless, no remarkable (*p* > 0.05) difference was apparently seen between the two cultivars.

**TABLE 2:** Varietal differences in fatty acid compositions of oils from ANKSE3 and UMA cultivars.

Component	ANKSE3	UMA
Palmitic acid (C16:0)	8.93^a^ ± 0.40	9.85^a^ ± 0.38
Palmitoleic acid (C16:1)	0.10^a^ ± 0.00	0.09^a^ ± 0.00
Stearic acid (C18:0)	5.91^a^ ± 0.04	4.95^a^ ± 0.00
Oleic acid (C18:1)	50.07^b^ ± 0.25	42.31^a^ ± 0.15
Linoleic acid (C18:2)	34.17^a^ ± 0.07	42.04^b^ ± 0.29
Linolenic acid (C18:3)	0.18^a^ ± 0.03	0.22^b^ ± 0.03
Arachidic acid (C20:0)	0.57^b^ ± 0.00	0.50^a^ ± 0.00
Behenic acid (C22:0)	0.08^a^ ± 0.28	0.06^a^ ± 0.00
∑Unsaturated FAs	84.52	84.66
∑Saturated FAs	15.41	15.36

*Note:* Each value in the table represents the mean of three replicates. Means within each row bearing different superscripts are significantly (*p* < 0.05) different.

### 3.3 DSC Thermal Profiles of *S. indicum* L. Oil

**FIGURE 2:**
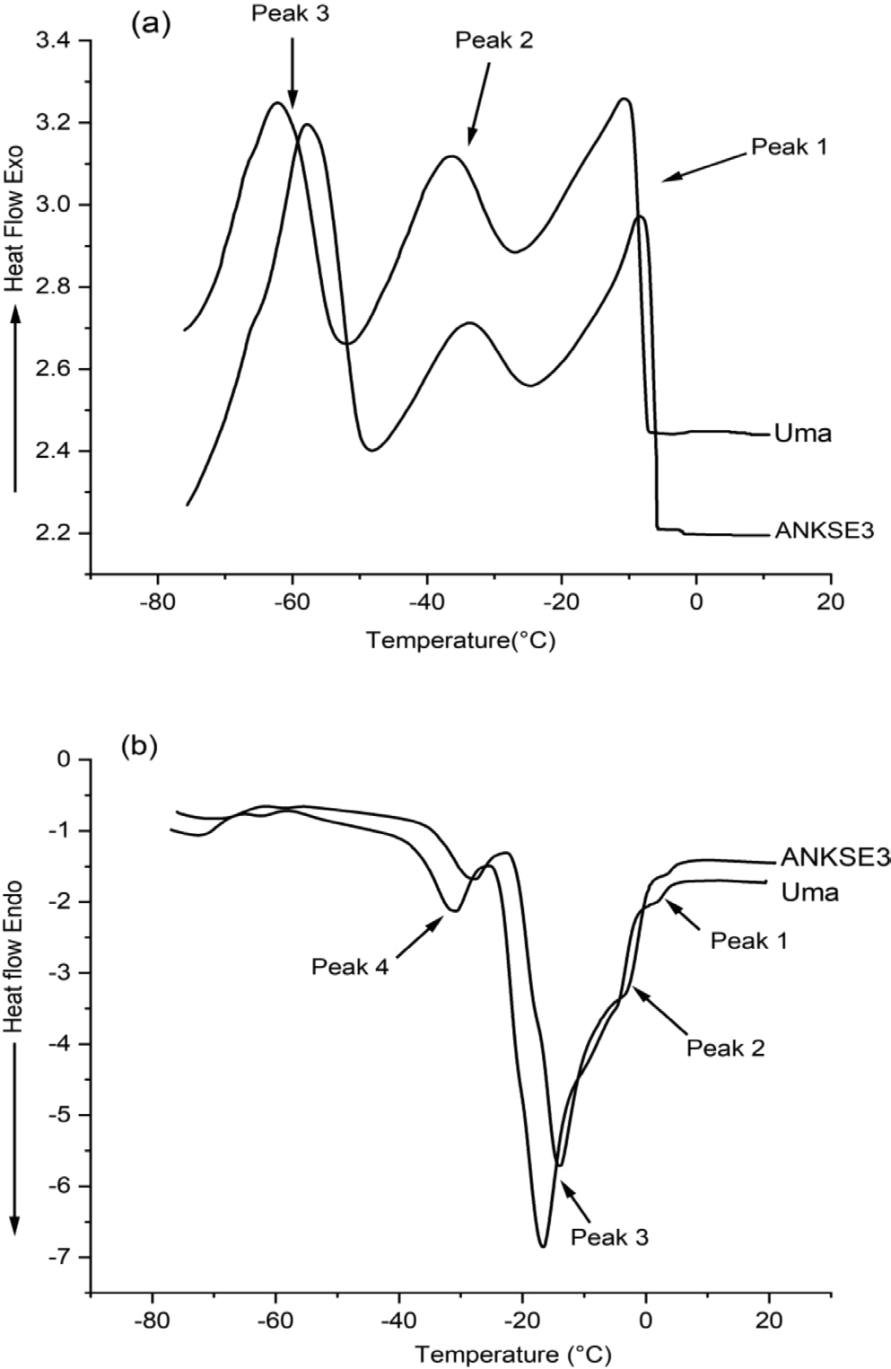
DSC cooling (a) and heating (b) curves of oils from ANKSE3 and UMA cultivars.

DSC cooling and heating curves of oils from ANKSE3 and UMA cultivars are presented in Figure 2a,b, respectively. The DSC cooling curve provides substantial information on thermal properties, including the cloud points of oils. According to thermal literature, cloud point is the temperature at which an oil sample sets to form a cloud, marking the initial stage of crystallization.

As shown in Figure 2a, three exothermic peaks were observed in the cooling curves. Based on the observations from Figure 2a, exothermic peak‐1 appeared in the high‐temperature region. Peak‐2 corresponds to the mid‐temperature region while peak‐3 corresponds to the low‐temperature region. Although the pattern of the cooling curves of the two cultivars was roughly similar, remarkable differences (*p* < 0.05) were observed with regard to certain DSC parameters corresponding to peaks 1, 2, and 3. The peak maxima of peak 1 in ANKSE3 and UMA were −8.21°C and −9.14°C, respectively. The peak areas of the major thermal transition (peak‐1) were 6.43 J/g for the ANKSE3 cultivar and 5.97 J/g for the UMA cultivar, with significant differences (*p <* 0.05) between them. Significant differences (*p <* 0.05) were also observed between the two cultivars in the onset temperatures of peak 1. The onset temperatures of oils from ANKSE3 and UMA were −5.64°C and −6.64°C, respectively, indicating the initiation of crystallization tends to begin earlier for the ANKSE3 cultivar. Additionally, the end‐set temperatures of ANKSE3 (−23.52°C) and UMA (−28.19°C) cultivars also showed remarkable (*p* < 0.05) differences between them.

All parameters of peak 2 exhibited remarkable differences (*p <* 0.05) between the two cultivars. For instance, peak maxima for ANKSE3 and UMA cultivars were recorded at −33.76°C and −36.24°C, respectively. Similarly, the onset temperature of the ANKSE3 cultivar was −26.24°C, while that of the UMA cultivar was −28.19°C. The peak areas of ANKSE3 and UMA cultivars were 2.48 and 3.12 J/g, respectively. Additionally, the end‐set temperatures for peak 2 were −47.47°C for the ANKSE3 cultivar and −53.67°C for the UMA cultivar with a remarkable difference (*p* < 0.05) between them.

With regard to parameters like peak maxima, onset temperature and peak area for peak 3, remarkable (*p* < 0.05) differences existed between the two cultivars. Nonetheless, no remarkable (*p* > 0.05) differences were noticed in end‐set temperatures of both cultivars. The peak maxima for ANKSE3 and UMA cultivars were −57.99°C and −61.33°C, respectively. Likewise, the onset temperatures of ANKSE3 and UMA cultivars were −50.39°C and −53.67°C, respectively. Nonetheless, the peak area of the ANKSE3 cultivar (10.68 J/g) was twice as high as that of the UMA cultivar (5.52 J/g), suggesting the highest energy release during the final stage of crystallization of the oil from the ANKSE3 cultivar.

DSC heating curves of ANKSE3 and UMA cultivars are shown in Figure 2b. DSC heating curves provide valuable information, including the melting point of an oil and phase transitions due to varied distribution of TAG groups. They also might help in visualizing deviations caused by oil adulterations. Based on visual observations, the heating curves of both cultivars were roughly similar. Major thermal transition (peak‐3) appearing in the mid‐temperature region is associated with peak‐1 and peak‐2 as shoulder peaks. A significant (*p* < 0.05) difference was seen between ANKSE3 and UMA cultivars with regard to the peak maxima of the major peak (Peak 3) at −14.27°C and −17.08°C, respectively. The peak area values of ANKSE3 and UMA cultivars were 20.13 and 21.14 J/g, respectively with a significant (*p* < 0.05) difference between them. Similarly, the onset temperatures of ANKSE3 and UMA cultivars were −19.14°C and −23.12°C, respectively. In addition, the end‐set temperatures of peak 3 for ANKSE3 and UMA cultivars were −7.38°C and −9.64°C, respectively. In fact, remarkable (*p* < 0.05) differences were observed between the two cultivars with regard to both onset and end‐set temperatures.

In the low‐temperature region, a minor thermal transition (peak‐4) was observed in both cultivars. This particular thermal transition was found to occur at −28.37°C and −31.46°C for ANKSE3 and UMA cultivars, respectively. The onset temperatures of peak‐4 were at −33.71°C for the ANKSE3 cultivar and −36.61°C for the UMA cultivar. Based on statistical analysis, remarkable differences (*p* < 0.05) were detected between the two cultivars regarding peak maxima and onset temperature. Similarly, the end‐set temperatures were −22.99°C for the ANKSE3 cultivar and −23.12°C for the UMA cultivar. The peak area values of ANKSE3 and UMA cultivars were 3.14 and 3.77 J/g, respectively, but no remarkable variation (*p* > 0.05) was noticed between the cultivars with regard to peak area and end‐set temperature.

### 3.4 FTIR Characterization of Sesame Oil From the Two Cultivars

**FIGURE 3:**
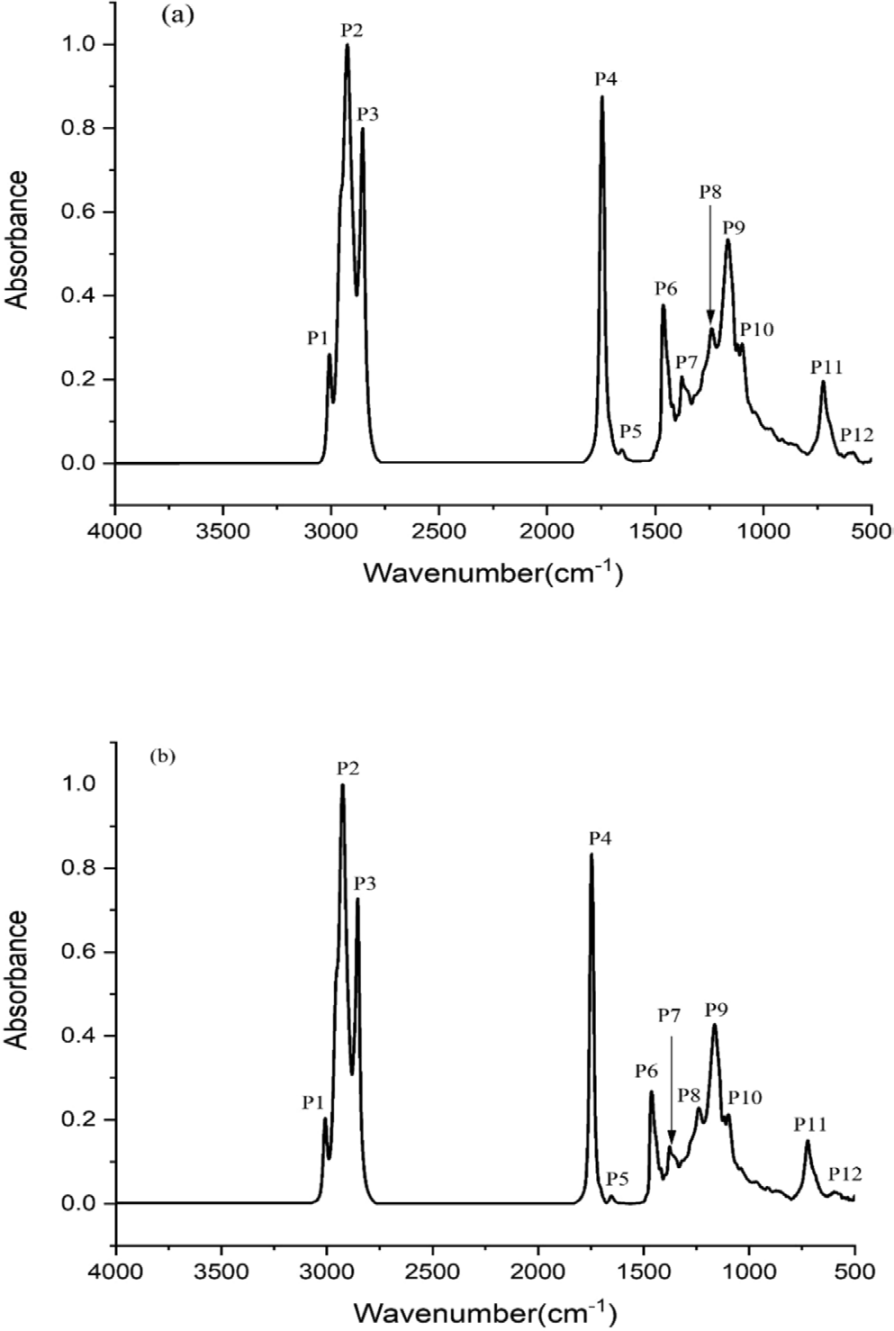
FTIR spectral overlay of oils from ANKSE3 (a) and UMA (b) cultivars.

Figure 3a,b illustrate the FTIR spectra of oils from ANKSE3 and UMA cultivars, respectively. According to Figure 3a,b, FTIR spectral patterns of the oils from the two cultivars were roughly similar. This similarity could be due to similar composition in triglycerides, which are the predominant component of fats and oils. The concrete bands and identification of functional groups were based on the previous findings reported in the literature. The peak 1 (P1) at ~3010 cm^−1^ corresponds to the terminal (vinyl) C—H stretching of alkenes. As shown in Figure 2, the distinguished peaks of P2 and P3 appearing at ~2925 and ~2855 cm^−1^ were caused by the asymmetrical and symmetrical C—H stretching of methylene groups, respectively. This was primarily due to the widespread occurrence of aliphatic chains in triacylglycerols. Further, the peak P4 appearing at ~1740 cm^−1^ was a distinctive sharp peak corresponding to the C=O stretching vibration of aliphatic ester moieties occurring in lipid biomolecules. This is a distinct oil peak commonly observed in most fats and oils, as they are composed of 98% triacylglycerol molecules. The blunt peak P5 at ~1655 cm^−1^ is usually assigned to C=C stretching vibration of alkenes.

Peaks in the fingerprint region are primarily suggestive of the presence of hydrocarbons and esters of oils and fats. For instance, the peak P6 observed at ~1455 cm^−1^ was associated with the asymmetric C—H bending of methyl groups, while P7 at ~1377 cm^−1^ resulted due to the symmetric C—H bending of hydrocarbons, validating the presence of alkanes (CH_3_) in the oils. The appearance of peak P8 at ~1238 cm^−1^ corresponds to the C—O stretching vibration of esters. The appearance of peaks P9 and P10 at ~1163 cm^−1^ and ~1100 cm^−1^ was caused by C—O stretching vibrations associated with esters or ethers. Furthermore, peak P11 found at ~722 cm^−1^ was attributed to (CH_2_)*n* bending vibration. The weak peak P12 appearing at ~585 cm^−1^, was due to C—S stretching of thiols, O—H bending of alcohol and C—I stretching of aliphatic iodo compounds.

### 3.5 Proximate Composition of Defatted Residues

Results of the proximate compositions of raw sesame seeds and defatted residues of the two cultivars are presented in Table 3. Fat was detected as the most dominant nutrient component in the raw sesame seeds of both cultivars, while protein was the most abundant nutrient component present in the defatted residues. The crude fat contents of raw sesame from the ANKSE3 and UMA cultivars were 42.01% and 43.75%, respectively. The crude fat content of defatted residues of ANKSE3 and UMA was 19.59% and 22.20%, respectively. It is clear that the fat contents of defatted residues from the two cultivars were approximately half that of the raw sesame.

**TABLE 3:** Proximate composition of whole sesame seed and defatted residues of ANKSE3 and UMA cultivars.

Parameters	Raw sesame seed		Defatted residue	
	ANKSE3	UMA	ANKSE3	UMA
Moisture (%)	4.77^b^ ± 0.03	4.36^a^ ± 0.12	6.96^B^ ± 0.07	5.76^A^ ± 0.66
Fat (%)	42.01^b^ ± 1.02	43.75^a^ ± 1.00	19.59^A^ ± 1.76	22.20^A^ ± 1.44
Protein (%)	18.99^a^ ± 0.42	19.84^a^ ± 0.48	34.45^A^ ± 0.47	36.1^A^ ± 0.33
Fiber (%)	6.97^a^ ± 0.33	5.67^a^ ± 0.40	9.1^B^ ± 0.43	7.245^A^ ± 0.40
Ash (%)	5.63^a^ ± 0.04	6.22^b^ ± 0.03	9.48^A^ ± 0.06	11.32^B^ ± 0.09
Total carbohydrate (%)	18.93^a^ ± 0.18	20.16^b^ ± 0.28	20.41^B^ ± 0.41	17.37^A^ ± 0.78

*Note:* Each value in the table represents the mean of three replicates ± standard deviation. Means that do not share a similar simple superscription letter in the same row in raw seeds and a similar capital superscription letter in the same row in defatted residue are significantly (*p* < 0.05) different.

Protein is the second most abundant macronutrient in sesame seeds. In fact, the oil extraction caused notable changes in protein, moisture, fiber and ash contents of defatted residues. According to Table 3, the protein content of raw sesame seeds ranged from 18.99% to 19.84%, while defatted residue ranged from 34.45% to 36.1%, where no remarkable (*p* > 0.05) difference was noticed in between the two cultivars. Moisture content of the defatted residues (5.76%–6.96%) was slightly higher than that of raw sesame seed (4.36%–4.77%). Likewise, the ash content of the defatted residues significantly (*p* < 0.05) increased (9.48%–11.32%) when compared to that of raw sesame seeds (5.63%–6.22%). The crude fiber content of raw sesame seed ranged from 5.67% to 6.97%, while defatted residues ranged from 7.24% to 9.1%, with a difference being statistically significant (*p* < 0.05) between the cultivars. Total carbohydrate refers to the sum of all types of carbohydrates, including dietary fiber, sugars, starches etc. The total carbohydrate content of the ANKSE3 cultivar (18.93% ± 0.18%) was lower than that of the UMA cultivar (20.16% ± 0.28%). Nonetheless, the results showed a notable increase in total carbohydrate content in defatted residues of ANKSE3 when compared to the raw seeds. Conversely, there was a significant decrease in the total carbohydrate content of defatted residue of UMA when compared to that of its raw sesame.

## 4 Discussion

### 4.1 Physicochemical Characteristics of *S. indicum* Oil

The color is an important quality parameter to ensure the freshness of vegetable oils. Two different types of pigments are generally responsible for the color of vegetable oils: naturally occurring lipid‐soluble pigments (carotenoids and chlorophylls) and process‐induced pigments, which are produced during oil processing (Chen and Sun 2023). Generally, the color of sesame oil might vary with cultivar differences and processing methods. According to the results of this study, the color of the UMA cultivar was more intense than that of the ANKSE3 cultivar probably due to the high content of carotenoids. Other than this, the roasting temperature might strongly influence the oil color due to Maillard reaction products. Owing to this reason, the color of the oil from unroasted sesame was yellow, while that of the roasted sesame was either brown or dark brown (Oboulbiga et al. 2023). Relatively, unroasted fresh sesame oil is slightly deeper in color when compared to coconut oil (SLS: 32 2017).

Sesame oil is generally found to have a higher IV when compared to coconut oil (7.8 g/100 g) and olive oil (84.1 g/100 g), indicating a higher degree of unsaturation because of its higher proportion of unsaturated FAs. A previous study by Hwang (2005) showed that the IV of sesame oil ranged from 104 to 120 g/100 g. According to another study, the IV for sesame oil is 106.6, which was due to the fact that, sesame oil contained roughly about 35% oleic acid and 47% linoleic acid (Kruatian and Jitmanee 2013). A high unsaturated FA content would make oils more prone to oxidation. Nevertheless, sesame oil contains a significant amount of antioxidants and vitamin E, which are meant to reduce oxidation. Oils that contain abundant mono and polyunsaturated fatty acids such as sesame oil are good at maintaining hypercholesterolemia conditions and reducing the risk of cardiovascular disease (Gouveia et al. 2017).

In any fat or oil, SV provides information about the average molecular weight of the constituent fatty acids. According to Borchani et al. (2010), SV of sesame oil was 186.6 mg KOH g^−1^, which was roughly similar to the results obtained in this study. Previous reports stated that a high SV for sesame oil is generally beneficial for cosmetics and skincare products due to its good emulsification ability (Wei et al. 2022). When compared to sesame oil, coconut oil was found to have a higher SV (248 mg of KOH g^−1^) (Kruatian and Jitmanee 2013).

### 4.2 Fatty Acid Profiles of *S. indicum* Oil

FA composition of sesame oil is vital in determining its nutritional and functional attributes. According to the data of this study, the oils consisted of approximately 84.5% USFAs and 15% SFAs. As mentioned before, oleic and linoleic acids were identified as the major USFAs in the two oils. These results are in agreement with the findings reported by Kurt (2018), who said that sesame oil from various countries was found to possess more than 80% USFAs, predominantly oleic and linoleic acids. This observation underscores the global uniformity in the FA profile of sesame oil, making it a reliable source of unsaturated FAs.

FA distributions of ANKSE3 and UMA cultivars differed significantly (*p* < 0.05), with notable variations in oleic and linoleic acid contents. By overall, their degree of unsaturation was roughly similar quantitatively. According to Saeed et al. (2015), black sesame oil was found to contain 50% oleic acid and 50% linoleic acid, while white sesame oil was found to contain 42% oleic acid and 43% linoleic acid. These findings were comparable to the studies reported in India by Thakur et al. (2018), where similar ranges were found for oleic and linoleic acids in both of the sesame cultivars. In a separate study, Agidew et al. (2021) reported that among Ethiopian sesame cultivars, linoleic acid was the major FA, followed by oleic, palmitic, and stearic acid. Furthermore, the amounts of linolenic and palmitoleic acids were reported to be less than 1%.

Linoleic is an essential FA required for various body functions, but the human body cannot synthesize it on its own, and must obtain it through food. It contributes to cholesterol metabolism by increasing the toughness of vascular epithelial cells, and supporting human growth and development (Wei et al. 2022). Furthermore, a diet rich in monounsaturated FAs (MUFA) helps prevent conditions like coronary heart disease (Reaven et al. 2015). Another study indicated that the proportion of palmitic acid (11%–21%) was comparatively higher than that of stearic acid (2%–5%) (Saeed et al. 2015). According to Agidew et al. (2021), the palmitic acid content ranged from 9% to 11%, while the stearic acid content ranged from 5% to 6%. Arachidic acid and behenic acid were detected in trace amounts, contributing less than 1% to the overall fatty acid profile. These findings aligned closely with the results of the present study.

FA profiles might vary significantly among oils, influencing their nutritional and functional properties. Coconut and palm kernel oils have displayed different FA profiles when compared to sesame oil. While sesame oil is known for higher linoleic acid, both coconut and palm kernel oils are notable for their high lauric acid content. According to Tan and Che Man (2000), coconut oil (51.1%) and palm kernel oil (55.8%) contain high lauric acid content but lower linoleic acid content compared to sesame oil. Hazelnut oil, canola and olive oils primarily consisted of oleic acid, accounting for 74.9%, 63%, and 72.5%, respectively. By overall, the variability in FA profiles underscores the diverse applications of these oils in nutrition and industry.

### 4.3 DSC Thermal Profiles of *S. indicum* Oil

As per the report by Tan and Che Man (2000), the cooling curve of sesame oil had exothermic peaks 1, 2, and 3, representing three primary TAG groups: monounsaturated (SSU), diunsaturated (SUU), and triunsaturated (UUU), respectively. These observations were attributed to the distribution of unsaturated fatty acids of sesame oil such as oleic acid (39%–43%) and linoleic acid (40%–44%) (Rajagukguk et al. 2022). On par with the cooling curves of sesame oil as reported by Tan and Che Man (2000), a pattern of three distinct and well‐defined peaks was in the thermal profile of this study. According to the previous study, DSC curves of sesame oil were found to display maximum temperatures of Peak 1, Peak 2, and Peak 3 as −14.09°C, −39.94°C and −66.62°C, respectively. These values differed considerably from those obtained in this study.

As indicated by this study, the crystallization temperature of sesame oil was comparatively lower than those of either coconut oil or palm oil. This could be due to the existence of an abundant amount of saturated FAs like lauric and palmitic acids in coconut and palm oils, which significantly affect the onset of crystallization (Tan and Che Man 2000). Similarly, canola and olive oils were found to display lower crystallization temperatures when compared to sesame oil. They were also reported to display three exothermic peaks with a distinctly sharp and tall peak. This is said to be due to the high oleic acid contents in canola and olive oils (Tan and Che Man 2000). In contrast to this, oils of ANKSE3 and UMA cultivars showed significantly different thermal curves, with differing peak areas and greater onset and peak maxima temperatures. This difference might be due to the complex composition or stronger interactions within their fatty acid profiles.

The findings of our study suggest that the melting points of the oils of ANKSE3 and UMA were at −14.27°C and −17.08°C, respectively. The observed difference in the melting temperature could be attributed to their differences in unsaturated fatty acids such as oleic acid (42%–50%) and linoleic acid (34%–42.0%) (Table 2). In the study of Tan and Che Man (2000), the temperature values of the major and minor peaks of pure sesame oil were detected at −21.45°C and −9.62°C, respectively. According to another sesame oil study by Rajagukguk et al. (2022), the major peaks of melting curves were at an average temperature of −19.93°C, while the minor peak was at −5.89°C. Their study, however, revealed that sesame oil recovered using hexane as a solvent and cold press extraction methods showed minor differences in their average melting peak temperatures. This minute variation could be due to differences in the compositions of the oils resulting from the different extraction methods. A study by Fahimdanesh et al. (2014) stated an endothermic (melting) criteria based on DCS for the authentication of sesame oil; the noted peak maxima of the major and minor peaks were −19.75°C and −34.92°C, respectively. According to this study, when sesame oil was mixed with three different concentrations of sunflower and corn oils, the peaks of the melting curves tended to shift to lower temperature regions. This movement was attributed to the increased proportion of saturated FAs in sunflower and corn oils (Fahimdanesh et al. 2014).

As mentioned previously, the DSC melting point values were relatively lower in sesame oil when compared to the coconut and palm kernel oils, which are rich in lauric acid (Tan and Che Man 2000). According to Tan and Che Man (2000), canola and olive oils exhibited a sharp, tall endothermic peak with shoulder peaks, showing a different pattern compared to sesame oil. Specifically, the melting behavior of oils from ANKSE3 and UMA cultivars was found to display a roughly similar pattern, but the thermal transitions of the ANKSE3 cultivar showed the highest onset, peak maxima and endset temperatures. The major melting peak at the higher temperature region indicated the presence of a high amount of unsaturated FAs like oleic and linoleic acids. The minor peak at the lower temperature region (peak‐4) highlighted the thermal behavior of low melting TAGs, including unsaturated and minor saturated FAs. According to Table 2, both of these cultivars displayed differences in unsaturated fatty acids such as oleic acid (42%–50%) and linoleic acid (34%–42.0%).

### 4.4 FTIR Spectral Characterization of *S. indicum* Oil

The FTIR study provides valuable information about the organic functional groups present in oil obtained from two local cultivars of sesame seed. Overall, the oils of ANKSE3 and UMA cultivars presented roughly similar spectral characteristics. Nonetheless, slight variations in peak intensities of the two cultivars were observed, especially within the fingerprint region (1500–950 cm^−1^). As a notable feature, the intensity of peaks within the fingerprint region was relatively higher for the ANKSE3 cultivar when compared to the UMA cultivar. Based on information from past literature, the presence of hydrocarbons and esters in the oils is indicated by the peaks that appear in the fingerprint region.

The most predominant peaks of the two oils namely P1 to P4 appeared within the 3010–1740 cm^−1^ wavenumber range. In both of the spectra, no peaks appeared within the 3600–3400 cm^−1^ range. This observation suggested that the oils are free from hydroxyl group (—OH), which are typically arising due to moisture, phenolic compounds or other hydrophilic substances. Interestingly, the spectrum of sesame oil presented a sharp peak P1 at ~3010 cm^−1^, which is also frequently observed in other unsaturated oils such as olive oil but is exhibited as a weak peak by coconut oil. It is agreed that the peak appearing in this region is mainly associated with the cis —C=C—H stretching, indicating the degree of unsaturation of the oils (Rohman 2017). According to this study, sesame oil contains about 85% of USFAs, with a high content of oleic and linoleic acids, which would have caused this peak (P1). Similar to sesame oil, the FTIR spectra of rapeseed and *Terminalia catappa* oils were also reported to display a sharp peak around ~3010 cm^−1^, suggesting the presence of USFAs in these oils (Halaldeen et al. 2024; Lu et al. 2014).

The prominent peaks P2 and P3 were due to the asymmetrical and symmetrical C—H stretching of methylene groups, while P4 was attributed to the ester carbonyl functional group of the triglycerides (Nandiyanto et al. 2023; Miller and Coates 2025). Previous study reports suggested that this distinct peak at ~1745 cm^−1^ was commonly found in the majority of the edible oils, including animal fats (Gunarathne et al. 2022). Further, the C=C stretching vibration of alkenes compounds is typically thought to be represented by the blunt peak P5 at ~1640 cm^−1^. Interestingly, this peak is absent in the FTIR spectrum of lauric oils but appears in other oils such as canola oil, olive oil, and rapeseed oil (Lu et al. 2014; Che Man and Rohman 2013; Rohman 2017). According to Mirghani et al. (2023), sesamol occurring in sesame oils may also be responsible for the stretching vibration at ~1636 cm^−1^. Based on previous research by Che Man and Rohman (2013), the distinguishing peak of this region was attributed to USFAs in some oils. In contrast to the spectra of some other vegetable oils, sesame oil did not exhibit a peak at ~962 cm^−1^, highlighting the rare occurrence of —HC=CH— (*trans*‐) double bond (Rohman 2017). The absence of *trans* double bonds in edible oils is advantageous in the context of food safety measures undertaken by regulatory authorities.

### 4.5 Proximate Composition of Defatted Residues

Sesame ( *S. indicum* L.) is known as an oil crop and its economic value is primarily due to high oil content. According to Perera et al. (2023), black sesame produced in Sri Lanka was found to have 48.46% oil. In a separate study, Singh et al. (2015) showed the crude oil contents of white sesame and black sesame cultivars were 47.79% and 48.21%, respectively. The sources of origin, variety, geographical location, etc. are generally attributed to observed variations in oil contents (Sabiha et al. 2020). Furthermore, in an exploratory study of the general nutritional composition of selected grains, Rajkumar and Selvakulasingam (2019) found that sesame had the highest crude fat content of 52.93%.

Sesame seed is generally considered a low‐moisture seed with a prolonged shelf life (Rajkumar and Selvakulasingam 2019). Ünal and Yalçın (2008) evaluated the moisture content of various sesame cultivars across different regions of Turkey; the range was between 4.16% and 4.62%. The findings reported were roughly similar to the results obtained in our study. Owing to its high content of protein, the defatted residue of sesame can be effectively utilized as a supplement in an extensive range of food products. It may be valuable for enhancing diets and addressing issues of protein‐malnutrition. For comparison purposes, however, there is hardly any literature on the proximate composition of defatted residues obtained from the ANKSE3 and UMA cultivars of Sri Lanka.

Sabiha et al. (2020) conducted a study on sesame cultivars of Pakistan; the ash content in defatted black and white sesame cultivars ranged from 4.73% to 6.33%, and 7.66% to 9.93%, respectively. The ash contents of the defatted residue of ANKSE3 and UMA cultivars were higher than those of Pakistani sesame cultivars, indicating a higher mineral content. Crude fiber refers to the portion of plant materials that are indigestible by humans. Based on our study, the defatted residue of sesame is a great source of dietary fiber, which can offer multiple health benefits such as promoting digestion and weight management, regulating blood sugar and cholesterol levels, improving gut health and reducing the risk of colon cancer. Being an abundant source of protein, ash and crude fiber, defatted residues of sesame will have good prospects as a high nutritional alternative plant protein source for value‐added formulations, considering sustainability and economic benefits.

## 5 Conclusion

This study demonstrated the feasibility of obtaining edible grade oil using a micro‐screw‐press expeller from two sesame cultivars without involving any chemical or solvent. The oil from the UMA cultivar exhibited higher values for color, iodine value, and saponification number when compared to the ANKSE3 cultivar. The high saponification number of sesame oil makes it particularly beneficial for cosmetics and skincare products. According to fatty acid profiling, the UMA cultivar contained higher linoleic acid content, while the ANKSE3 cultivar contained higher oleic acid content. Hence, the oil of the UMA cultivar could be utilized in dietary supplements due to the abundance of essential fatty acids. With regard to DSC thermal profiles, sesame oils display lower melting and crystallization points. This property is particularly beneficial to use as a salad oil. The FTIR spectral analysis of sesame oils from both ANKSE3 and UMA cultivars showed a similar pattern for organic functional groups, with alkenes, alkanes, esters, and hydrocarbons being the major ones. According to proximate analysis, the oil extraction caused drastic changes in all parameters of the defatted residues; sesame seeds are rich in oil, but their defatted residues are high in protein content. Particularly, the defatted residue of the UMA cultivar has shown significantly higher contents of protein and ash, while the ANKSE3 cultivar indicated higher contents of crude fiber and carbohydrates. Overall, the distinct physicochemical properties and nutritional composition of the ANKSE3 and UMA cultivars and physicochemical characteristics of the oils highlighted their potential applications in food and dietary supplements.

## Author Contributions

**Sazna Fariz:** formal analysis (equal), methodology (equal), software (equal), writing – original draft (equal). **Fahmidha Halaldeen:** data curation (equal), formal analysis (equal), investigation (equal). **Terrance Madhujith:** conceptualization (equal), supervision (equal). **Nazrim Marikkar:** conceptualization (equal), project administration (equal), supervision (equal), writing – review and editing (equal). **Muneeb M. Musthafa:** project administration (equal), validation (equal), visualization (equal), writing – review and editing (equal). **Mohammed Arshad:** project administration (equal), writing – original draft (equal), writing – review and editing (equal). **Abdul Aziz Al Kheraif:** project administration (equal), validation (equal), writing – review and editing (equal).

## Conflicts of Interest

The authors declare no conflicts of interest.

## Data Availability

The data supporting this study's findings are available from the corresponding author upon request.
